# Oxygen modulates iron homeostasis by switching iron sensing of NCOA4

**DOI:** 10.1016/j.jbc.2023.104701

**Published:** 2023-04-13

**Authors:** Sota Kuno, Kazuhiro Iwai

**Affiliations:** Department of Molecular and Cellular Physiology, Graduate School of Medicine, Kyoto University, Kyoto, Japan

**Keywords:** autophagy, ferritin, hypoxia, iron metabolism, iron–sulfur protein

## Abstract

To ensure proper utilization of iron and avoid its toxicity, cells are equipped with iron-sensing proteins to maintain cellular iron homeostasis. We showed previously that nuclear receptor coactivator 4 (NCOA4), a ferritin-specific autophagy adapter, intricately regulates the fate of ferritin; upon binding to Fe^3+^, NCOA4 forms insoluble condensates and regulates ferritin autophagy in iron-replete conditions. Here, we demonstrate an additional iron-sensing mechanism of NCOA4. Our results indicate that the insertion of an iron–sulfur (Fe–S) cluster enables preferential recognition of NCOA4 by the HERC2 (HECT and RLD domain containing E3 ubiquitin protein ligase 2) ubiquitin ligase in iron-replete conditions, resulting in degradation by the proteasome and subsequent inhibition of ferritinophagy. We also found that both condensation and ubiquitin-mediated degradation of NCOA4 can occur in the same cell, and the cellular oxygen tension determines the selection of these pathways. Fe–S cluster–mediated degradation of NCOA4 is enhanced under hypoxia, whereas NCOA4 forms condensates and degrades ferritin at higher oxygen levels. Considering the involvement of iron in oxygen handling, our findings demonstrate that the NCOA4–ferritin axis is another layer of cellular iron regulation in response to oxygen levels.

Iron is an essential trace element that functions as a cofactor in a wide variety of biological processes, such as oxygen delivery, ATP production, and DNA synthesis and repair, by binding to proteins (1, 2). However, excess iron is toxic owing to its ability to promote the generation of reactive oxygen species (3, 4). Ferroptosis (5), a type of regulated cell death caused by iron-induced lipid peroxidation, is a well-known outcome of iron toxicity (6, 7). Therefore, organisms are equipped with sophisticated iron-sensing mechanisms that ensure tight control of the cellular iron homeostasis.

In mammals, the cytosolic iron storage protein ferritin plays a central role in maintaining iron homeostasis (8). Ferritin forms cages comprising 24 light (FTL) and heavy (FTH) subunits and can store up to some 3000 ferric iron (Fe^3+^) ions (9). In iron-deficient conditions, the production of ferritin is suppressed at the post-transcriptional level by iron regulatory proteins 1 and 2 (IRP1 and IRP2) (10, 11, 12). Mechanistically, IRPs inhibit translational initiation by binding to a stem–loop structure in the ferritin mRNA known as the iron-responsive element (IRE). Under iron-replete conditions, IRPs lose their ability to bind the IRE, leading to increased ferritin production. IRPs also upregulate production of the iron-uptake protein transferrin receptor 1 (TfR1) in iron-depleted conditions and suppress its production in iron-sufficient conditions. Hence, IRPs have been recognized as central regulators of cellular iron metabolism.

The amount of ferritin is also regulated by autophagy. When cells are subjected to iron depletion, ferritin is delivered rapidly to lysosomes, where it is degraded *via* canonical macroautophagy to release the stored iron (13). Nuclear receptor coactivator 4 (NCOA4) is a ferritin-specific selective autophagy adapter that ensures a rapid decrease in the level of ferritin in response to iron deficiency (14, 15). We reported recently that the amount of ferritin is also regulated by NCOA4 in iron-replete conditions (16). In this context, binding of Fe^3+^ to the intrinsically disordered region (IDR) of NCOA4 induces condensation of the protein. The condensates are sequestered away from ferritin in the early phase of iron repletion, allowing ferritin to be accumulated for storage of excess iron and the maintenance of iron homeostasis. In prolonged iron-replete conditions, to prevent iron deficiency caused by excessive iron storage and reduced iron uptake by TfR1, NCOA4 condensates promote the delivery of ferritin to lysosomes. Therefore, NCOA4 functions as a crucial regulator of iron metabolism by coordinating with the IRP system to fine-tune the fate of ferritin (16). However, a previous study reported that excess iron induces recognition of NCOA4 by the HERC2 (HECT and RLD domain containing E3 ubiquitin protein ligase 2) ubiquitin ligase and its subsequent ubiquitin-mediated degradation, a process that ensures ferritin accumulation in iron-rich conditions (17).

Here, we examined whether iron-dependent NCOA4 condensation is a common mechanism that regulates the fate of ferritin in iron-replete cells. We found that NCOA4 forms insoluble condensates in most human cell lines upon iron administration, whereas it is degraded by HERC2-mediated ubiquitination in human embryonic kidney 293T (HEK293T) cells. Our results demonstrate that HERC2 preferentially recognizes NCOA4 harboring an Fe–S cluster, leading to degradation of NCOA4 and subsequent accumulation of ferritin. A NCOA4 mutant lacking the Fe–S cluster insertion formed condensates in HEK293T cells, suggesting that both Fe^3+^-mediated NCOA4 condensation and Fe–S-mediated degradation of NCOA4 can occur in the same cell. Furthermore, we found that the oxygen level is a crucial determinant of NCOA4 regulation; whereas lower oxygen levels facilitate loading of the Fe–S cluster onto NCOA4 and its subsequent degradation, higher oxygen levels induce iron-dependent condensation of NCOA4. Our study provides novel insights into iron-sensing mechanisms that regulate the fate of ferritin as well as the relationship between iron metabolism and oxygen levels.

## Results

### Iron-dependent regulation of NCOA4 is cell type specific

**Figure 1:**
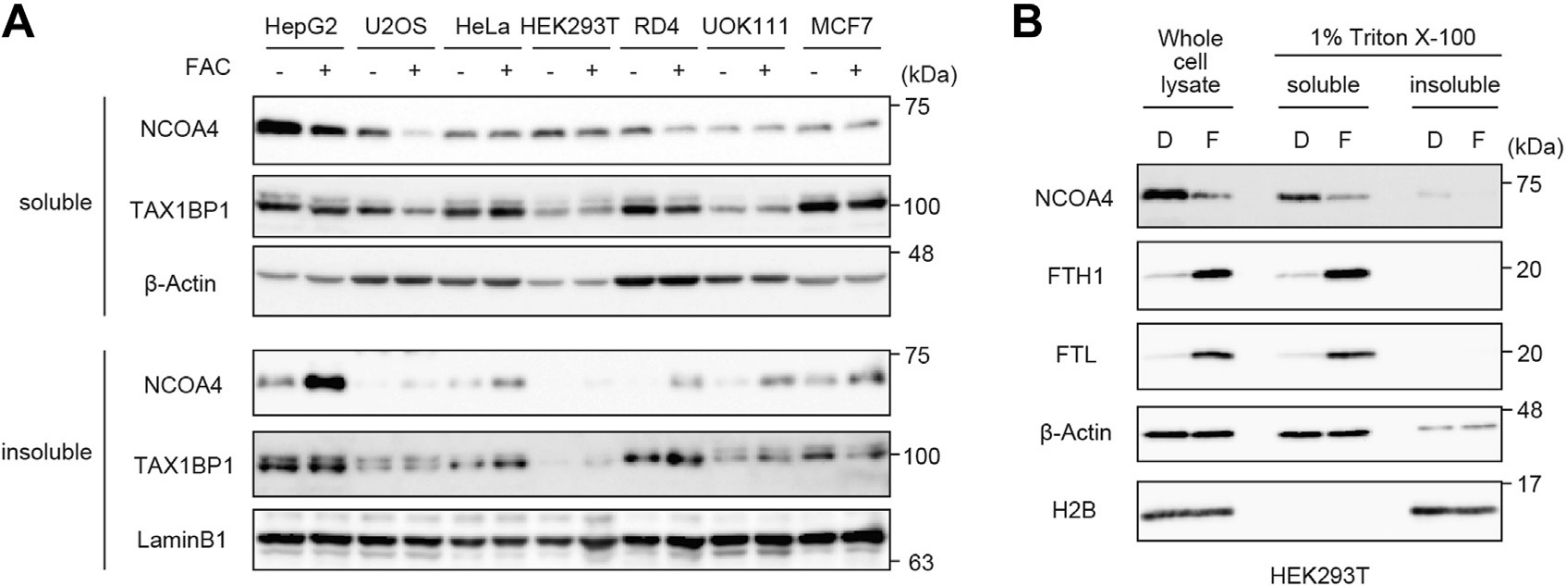
**Iron-dependent regulation of NCOA4 differs between cell types.***A*, immunoblot analyses of NCOA4, TAX1BP1, and β-actin in seven human cell lines treated with or without 25 μg/ml FAC for 12 h. The soluble and insoluble fractions in Triton buffer were subjected to SDS-PAGE and analyzed by immunoblotting with the indicated antibodies. *B*, immunoblot analyses of NCOA4, FTH1, FTL, β-actin, and H2B in HEK293T cells treated with 20 μM Dfo (D) or 25 μg/ml FAC (F) for 3 h. The cells were lysed with SDS sample buffer or Triton buffer and analyzed by immunoblotting with the indicated antibodies. Dfo, deferoxamine; FAC, ferric ammonium citrate; HEK293T, human embryonic kidney 293T cell line.

**Supporting Figure S1:**
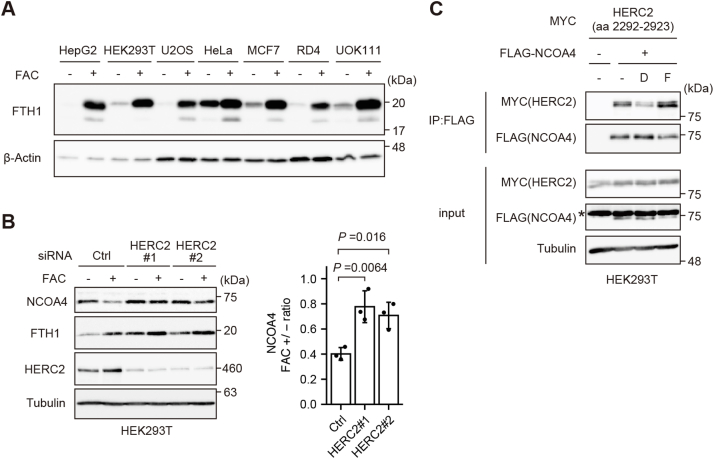
**Iron-dependent regulation of NCOA4 is cell type-specific.***A*, immunoblot analyses of FTH1 and β-actin in seven human cell lines treated with or without 25 μg/ml FAC for 12 h. Soluble fractions in Triton buffer were subjected to SDS-PAGE and analyzed by immunoblotting with an anti-FTH1 antibody. *B*, immunoblot analyses of NCOA4, FTH1, HERC2, and β-actin in HEK293T cells transfected with control or HERC2-specific siRNAs for 2 days. The cells were treated with 25 μg/ml FAC for 2 h and then harvested. Soluble cell lysates were subjected to SDS-PAGE and analyzed by immunoblotting with the indicated antibodies. *P*-values were calculated *via* one-way ANOVA with Dunnett post hoc test. *C*, the effect of iron treatment or chelation on the interaction between NCOA4 and HERC2 in HEK293K cells. The cells were transiently transfected with FLAG-NCOA4 WT and myc-HERC2 (amino acids 2292–2923), cultured for 2 days, and then treated with 20 μM Dfo (D) or 10 μg/ml FAC (F) for 6 h. Cell lysates were immunoprecipitated with an anti-FLAG antibody, and the amount of coimmunoprecipitated myc-HERC2 was evaluated by immunoblotting. The *asterisk* indicates non-specific bands.

To determine whether iron-induced condensation of NCOA4 is a common mechanism determining the fate of ferritin, we first examined the effect of the cellular iron status on NCOA4 expression in seven human cell lines. The addition of ferric ammonium citrate (FAC) to the culture medium increased the amount of ferritin (FTH1) in all cell lines tested (Fig. S1*A*). In six of the seven iron-treated cell lines, the majority of NCOA4 was found in the insoluble fraction, suggesting iron-dependent condensation (Fig. 1*A*) (16). By contrast, NCOA4 was present mainly in the soluble fraction of HEK293T cells, although FAC treatment reduced the amount in this fraction. Lysis of HEK293T cells with SDS buffer or Triton X-100 buffer revealed that the total amount of NCOA4 was lower in FAC-treated cells than in iron-chelated cells treated with deferoxamine (Fig. 1*B*). As mentioned previously, a previous report suggested that NCOA4 is recognized by the HERC2 ubiquitin ligase and subsequently degraded by the proteasome (17). Here, siRNA-mediated knockdown of HERC2 inhibited the iron-dependent reduction in NCOA4 expression (Fig. S1*B*). Overall, these results indicate that iron treatment promotes the formation of insoluble NCOA4 condensates in most cell lines tested, with the exception of HEK293T cells.

We showed previously that insoluble NCOA4 condensates deliver ferritin to lysosomes *via* a noncanonical autophagy pathway under prolonged iron repletion (16). Consistent with this finding, a faster migrating band of ferritin, representing a partial lysosomal degradation product (13, 14, 16), was seen in the six cell lines in which NCOA4 was detected in the insoluble fraction but was not seen in HEK293T cells (Fig. S1*A*). Therefore, the fate of NCOA4 is differentially regulated by iron in a cell type–specific manner.

### Possible involvement of the Fe–S cluster in HERC2-mediated degradation of NCOA4

**Figure 2:**
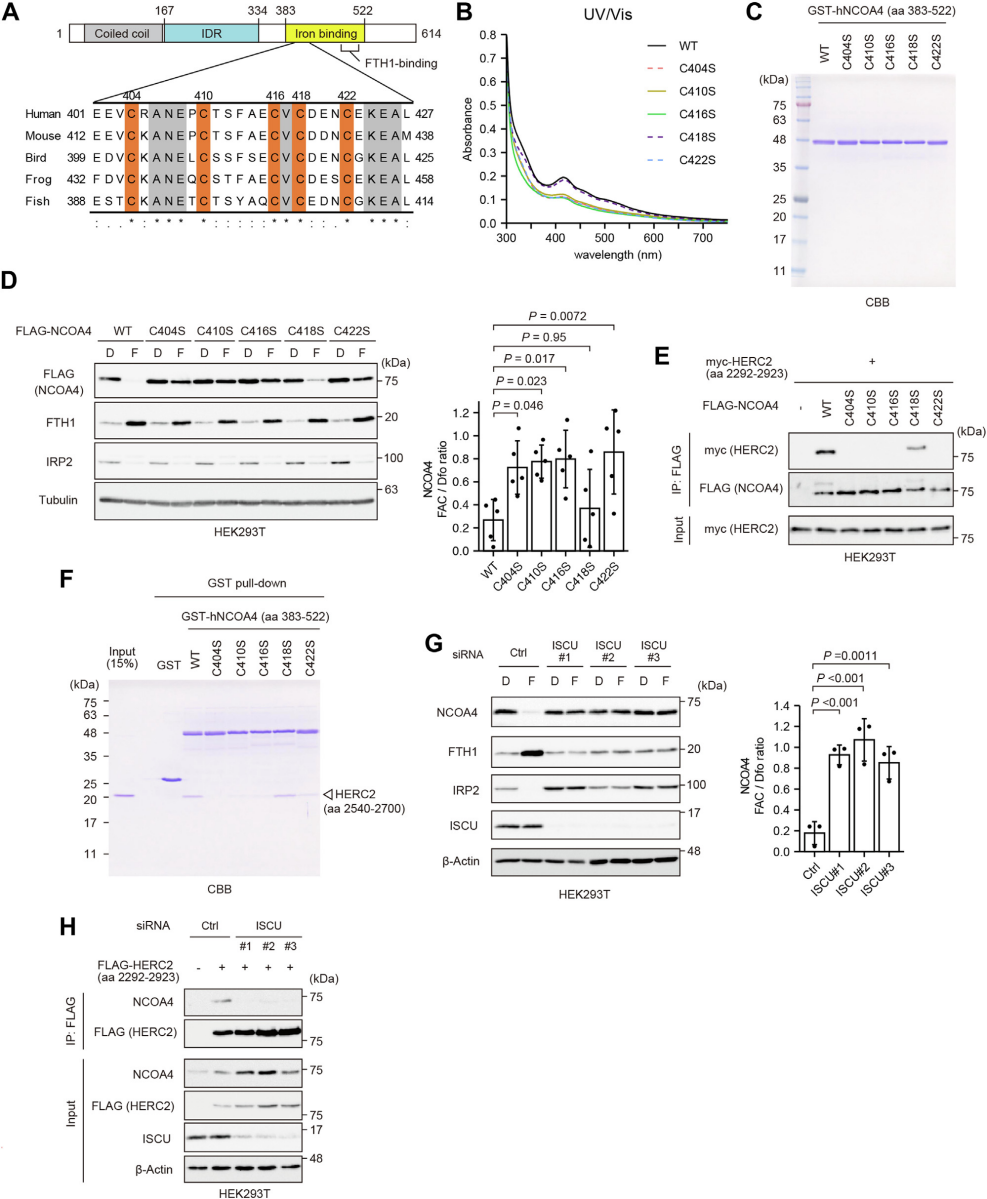
**HERC2 preferentially recognizes and degrades Fe–S cluster–ligated NCOA4.***A*, sequence alignment of the Cys-rich region in NCOA4 orthologs from human (*Homo sapiens*), mouse (*Mus musculus*), bird (*Gallus gallus*), frog (*Xenopus tropicalis*), and fish (*Danio rerio*). Conserved Cys residues are highlighted in *orange*. Strictly conserved residues are highlighted in *gray*. *B*, the UV–visible absorption spectra of the purified proteins shown in (*C*). The protein concentration was 2 mg/ml. *C*, SDS-PAGE analysis of purified WT and mutant GST-NCOA4 (amino acids 383–522) proteins (1 μg/lane). The gel was stained with Coomassie brilliant blue (CBB). *D*, immunoblot analyses of FLAG-NCOA4, FTH1, IRP2, and β-actin in HEK293T cells transiently transfected with WT or mutant FLAG-NCOA4, cultured for 2 days, and then treated with 20 μM Dfo (D) or 25 μg/ml FAC (F) for 3 h. Soluble cell lysates were subjected to SDS-PAGE and analyzed by immunoblotting with the indicated antibodies. *E*, immunoblot analyses of myc-HERC2 and FLAG-NCOA4 in HEK293T cells transiently transfected with WT or mutant FLAG-NCOA4 and myc-HERC2 (amino acids 2292–2923), cultured for 2 days, and then treated with 25 μg/ml FAC for 3 h. Cell lysates were immunoprecipitated with an anti-FLAG antibody. The amount of coimmunoprecipitated myc-HERC2 was evaluated by immunoblotting. *F*, GST pulldown assay using recombinant WT and mutant GST-NCOA4 (amino acids 383–522) and His-HERC (amino acids 2540–2700). The gel was stained with CBB. *G*, immunoblot analyses of NCOA4, FTH1, IRP2, ISCU, and β-actin in HEK293T cells treated with control or ISCU-specific siRNAs for 2 days, and then treated with 20 μM Dfo (D) or 25 μg/ml FAC (F) for 3 h. Soluble cell lysates were subjected to SDS-PAGE and analyzed by immunoblotting with the indicated antibodies. *H*, immunoblot analyses of NCOA4, FLAG-HERC2, ISCU, and β-actin in HEK293T cells cotransfected with control or ISCU-specific siRNAs and FLAG-hHERC2 (amino acids 2292–2923) or FLAG empty vector. The cells were cultured for 2 days and then treated with 25 μg/ml FAC for 3 h. Cell lysates were immunoprecipitated with an anti-FLAG antibody. The amount of coimmunoprecipitated endogenous NCOA4 was evaluated by immunoblotting. *D* and *G*, *p* Values were calculated *via* one-way ANOVA with Dunnett post hoc test. Dfo, deferoxamine; FAC, ferric ammonium citrate; Fe–S, iron–sulfur; GST, glutathione *S*-transferase; HEK293T, human embryonic kidney 293T cell line; IRP, iron regulatory protein.

**Supporting Figure S2:**
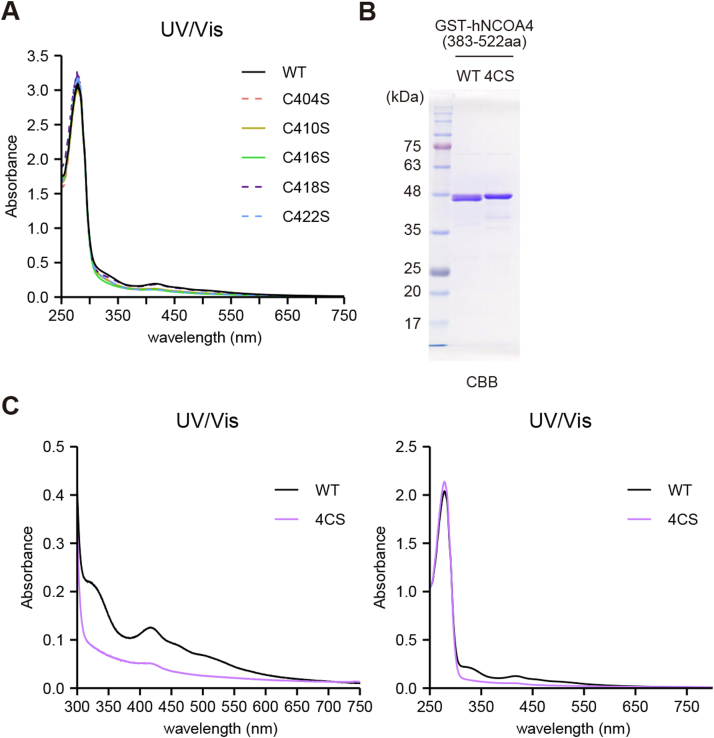
**NCOA4 can likely possess an Fe-S cluster.***A*, overall picture of the UV-visible absorption spectra of the purified proteins shown in Figure 2*B*. *B*, SDS-PAGE analysis of the WT and 4CS mutant GST-NCOA4 (amino acids 383–522) proteins (1 μg/lane). The gel was stained with Coomassie brilliant blue (CBB). *C*, The UV-visible absorption spectra of the purified proteins shown in (*B*). The protein concentration was 1.3 mg/ml.

To probe the mechanisms underlying the two modes of regulation of NCOA4 under iron-replete conditions, we examined the regulation of NCOA4 by HERC2 in more detail. A previous study showed that upon binding to iron, amino acids 383 to 522 of NCOA4 are preferentially recognized by HERC2 (17). Here, as reported in the previous study (17), an immunoprecipitation experiment confirmed that the HERC2 fragment spanning amino acids 2292 to 2923 bound NCOA4 more efficiently in iron-replete conditions than in iron-chelated conditions (Fig. S1*C*). We showed previously that binding of Fe^3+^ to the IDR (amino acids 167–334) of NCOA4 promotes condensation of the protein. Thus, to investigate what form of iron binds to amino acids 383 to 522 of NCOA4 to enable its recognition by HERC2, we expressed and purified a glutathione *S*-transferase (GST)-fused NCOA4 (amino acids 383–522) protein using a bacterial expression system. UV–visible spectrometry of GST-NCOA4 (amino acids 383–522) showed peaks at 330 and 420 nm as well as a broad shoulder at a longer wavelength (*black solid line* in Figs. 2*B* and S2*A*), a spectrum that is similar to that of 2Fe–2S cluster proteins (18). Regardless of the specific type, Fe–S cluster proteins generally ligate the cluster *via* four cysteine (Cys) or histidine residues (18). An *in silico* analysis revealed that five Cys residues in human NCOA4 (amino acids 383–522) are conserved among NCOA4 orthologs (Fig. 2*A*). UV–visible spectrometry analyses of GST-NCOA4 (amino acids 383–522) mutants in which one of the five Cys residues was substituted with serine revealed that the C404S, C410S, C416S, and C422S mutants exhibited attenuated absorption at 330 and 420 nm, whereas the C418S mutant did not (Figs. 2, *B* and *C* and S2*A*). Furthermore, simultaneous mutation of C404, C410, C416, and C422 to serine abrogated the characteristic spectrum almost completely (Fig. S2, *B* and *C*). Collectively, these results suggest that iron binds to the HERC2 binding region (amino acids 383–522) of NCOA4 as an Fe–S cluster.

To examine their cellular fate, the NCOA4 mutants were expressed exogenously in HEK293T cells. The levels of the four NCOA4 mutants that were unable to bind an Fe–S cluster (C404S, C410S, C416S, and C422S) were not decreased upon FAC treatment, and these mutants did not interact with the HERC2 (amino acids 2292–2923) fragment (Fig. 2, *D* and *E*). In addition, an *in vitro* binding assay using purified GST-NCOA4 (amino acids 383–522) mutants and HERC2 (amino acids 2540–2700), as used in a previous study (17), revealed that, with the exception of C418S, the mutants failed to interact with HERC2 (amino acids 2540–2700) (Fig. 2*F*).

Next, we investigated the fate of NCOA4 following the disruption of Fe–S cluster biogenesis. The generation of Fe–S clusters is initiated in mitochondria, and the first step of biogenesis involves the scaffold protein ISCU (19, 20, 21). The siRNA-mediated knockdown of ISCU inhibited the degradation of NCOA4 in FAC-treated HEK293T cells almost completely (Fig. 2*G*). In addition, knockdown of ISCU affected Fe–S synthesis, as indicated by an increase in the amount of IRP2, which is recognized by 2Fe–2S ligated FBXL5 in the SCF^FBXL5^ ubiquitin ligase complex and is destined for degradation in iron-replete conditions (22). Furthermore, the amount of ferritin was reduced on account of the stabilization of IRP2. We also found that knockdown of ISCU abrogated the interaction between endogenous NCOA4 and an ectopically expressed HERC2 fragment (amino acids 2292–2923) (Fig. 2*H*). Taken together, these results suggest that HERC2 preferentially recognizes and degrades NCOA4 harboring an Fe–S cluster.

### NCOA4 forms insoluble condensates and degrades ferritin upon inhibition of Fe–S insertion in iron-treated HEK293T cells

**Figure 3:**
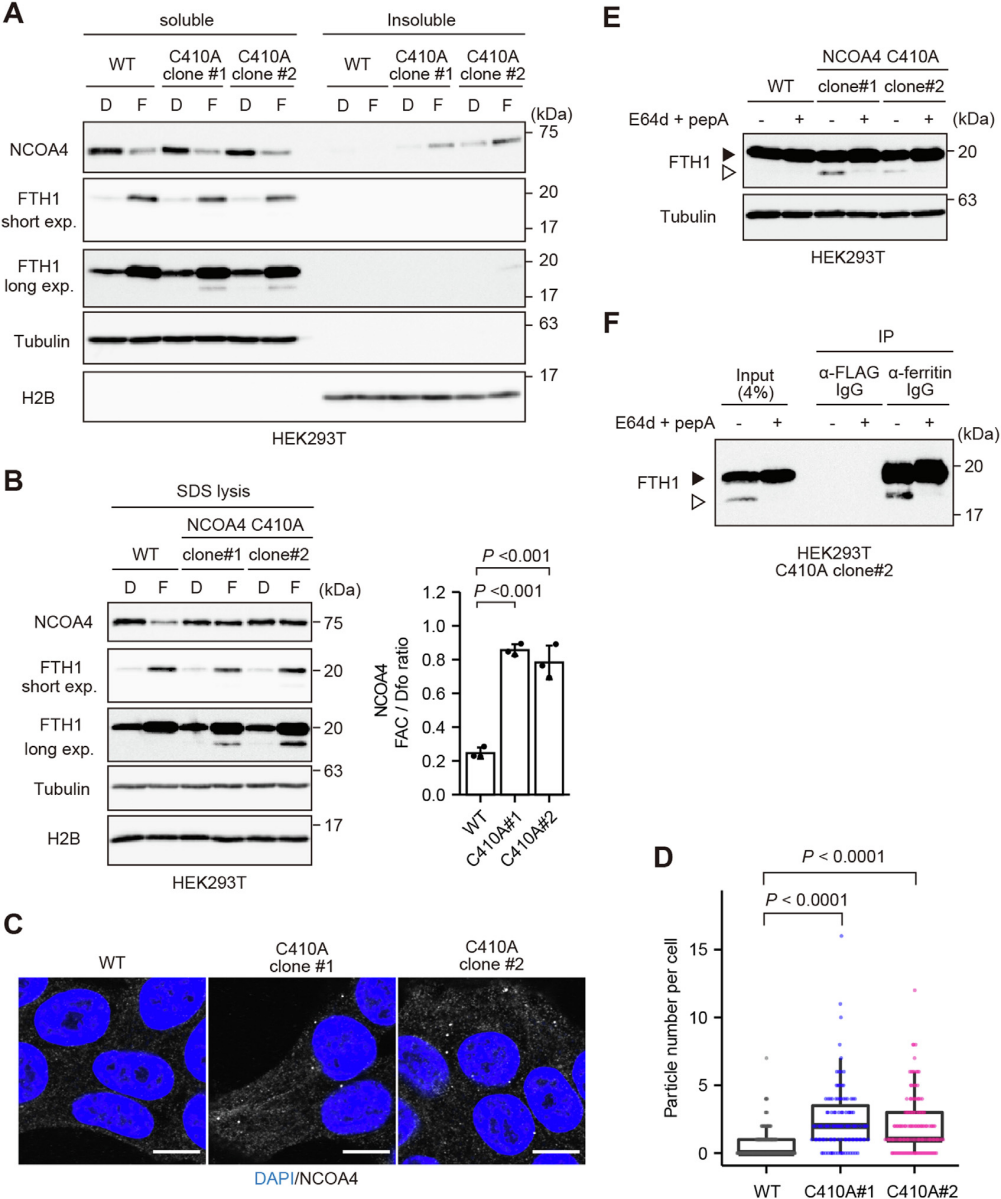
**NCOA4 forms insoluble condensates and degrades ferritin upon inhibition of Fe–S insertion in iron-treated HEK293T cells.***A* and *B*, immunoblot analyses of NCOA4, FTH1, tubulin, and H2B in WT HEK293T cells and prime-edited HEK293T cells expressing endogenous NCOA4 C410A, following treatment with 20 μM Dfo (D) or 25 μg/ml FAC (F) for 3 h. Cells were lysed with Triton buffer (*A*) or SDS-containing buffer (*B*). The lysates were subjected to SDS-PAGE and analyzed by immunoblotting with the indicated antibodies. *p* Values were calculated *via* one-way ANOVA with Dunnett post hoc test. *C*, fluorescence microscopy images of WT and NCOA4 C410A HEK293T cells, following treatment with 25 μg/ml FAC for 3 h. The cells were stained with an anti-NCOA4 antibody and DAPI. Representative images are shown. Scale bar represents 10 μm. *D*, quantitative analysis of the particle numbers in the cells described in (*C*). At least 100 cells were quantified from three individual experiments. A Kruskal–Wallis ANOVA with Dunn’s multiple comparison test was performed. *p* Values were less than 0.0001 and adjusted using the Bonferroni method. *E* and *F*, immunoblot analyses of FTH1 and tubulin in WT and NCOA4 C410A HEK293T cells (*E*) or FTH1 in NCOA4 C410A HEK293T cells (clone #2) (*F*), following treatment with 25 μg/ml FAC and either DMSO or 10 μg/ml E64d and 10 μM pepstatin A (pepA) for 12 h. *E*, soluble lysates in Triton buffer were subjected to SDS-PAGE. *F*, soluble cell lysates were immunoprecipitated with an anti-FLAG or anti-ferritin antibody. Soluble lysates and immunoprecipitates were subjected to SDS-PAGE. The *black arrowhead* indicates intact FTH1, and the *white arrowhead* indicates partially degraded FTH1. DAPI, 4′,6-diamidino-2-phenylindole; Dfo, deferoxamine; DMSO, dimethyl sulfoxide; FAC, ferric ammonium citrate; Fe–S, iron–sulfur; HEK293T, human embryonic kidney 293T cell line.

**Supporting Figure S3:**
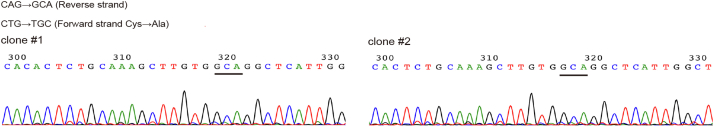
**Confirmation of the C410A mutation of NCOA4 in HEK293T cells.** Sanger sequencing of NCOA4 in prime-edited HEK293T cells.

As described previously, recognition by HERC2 results in the degradation of NCOA4 and subsequent accumulation of ferritin in iron-replete HEK293T cells (Fig. 2). However, we demonstrated previously that, upon binding of Fe^3+^ to its IDR, NCOA4 forms insoluble condensates in mouse embryonic fibroblasts (MEFs) and other cell lines (Fig. 1*A*) (16). Therefore, we examined whether inhibiting insertion of the Fe–S cluster into NCOA4 would result in the formation of solid-like condensates in iron-treated HEK293T cells. To this end, we intended to mutate one of the four Cys residues involved in Fe–S ligation (Fig. 2). In view of a technical limitation of the prime editing system, we selected Cys410 and generated HEK293T cells expressing an endogenous NCOA4 mutant in which Cys410 was replaced by alanine (C410A) (Fig. S3) (23, 24). In contrast to the overexpression experiment shown in Figure 2, in which the iron-induced decrease in the level of overexpressed NCOA4 was abolished by the C410S mutation, the C410A mutation in endogenous NCOA4 did not overtly affect the iron-induced decrease in the level of NCOA4 in the soluble fraction; however, NCOA4 C410A, but not WT NCOA4, was accumulated in the insoluble fraction (Fig 3*A*). Lysis of the cells with SDS lysis buffer confirmed that iron treatment reduced the total amount of WT NCOA4 but did not affect that of NCOA4 C410A (Fig. 3*B*). These results indicate that disrupting the insertion of an Fe–S cluster into NCOA4 does not alter the total amount of endogenous NCOA4 in HEK293T cells but does alter its biochemical characteristics to match those of WT NCOA4 in MEFs and other cell lines (Fig. 1*A*) (16).

Consistent with our previous observation that NCOA4 in the insoluble fraction forms dot-like structures (16), endogenous prime-edited NCOA4 C410A formed condensates in HEK293T cells following iron administration (Fig. 3, *C* and *D*). A faster migrating FTH1 band was observed in immunoblot analyses of iron-replete HEK293T cells expressing endogenous NCOA4 C410A (Fig. 3, *A* and *B*). Immunoprecipitation and immunoblotting analyses using two different anti-FTH1 antibodies confirmed that the faster migrating band was partially degraded ferritin in the lysosomes, as reported in previous studies (13, 14, 16). This faster migrating band disappeared following treatment of NCOA4 C410A HEK293T cells with the lysosomal protease inhibitors E64d and pepstatin A (Fig. 3, *E* and *F*). These findings indicate that insoluble NCOA4 condensates deliver ferritin to lysosomes upon inhibition of Fe–S cluster insertion in HEK293T cells. Overall, these results demonstrate that both Fe–S cluster–mediated NCOA4 degradation and iron-induced NCOA4 condensation can operate in iron-replete HEK293T cells, but the former seems to predominate.

### Noncanonical ferritinophagy alters cellular iron metabolism in HEK293T cells

**Figure 4:**
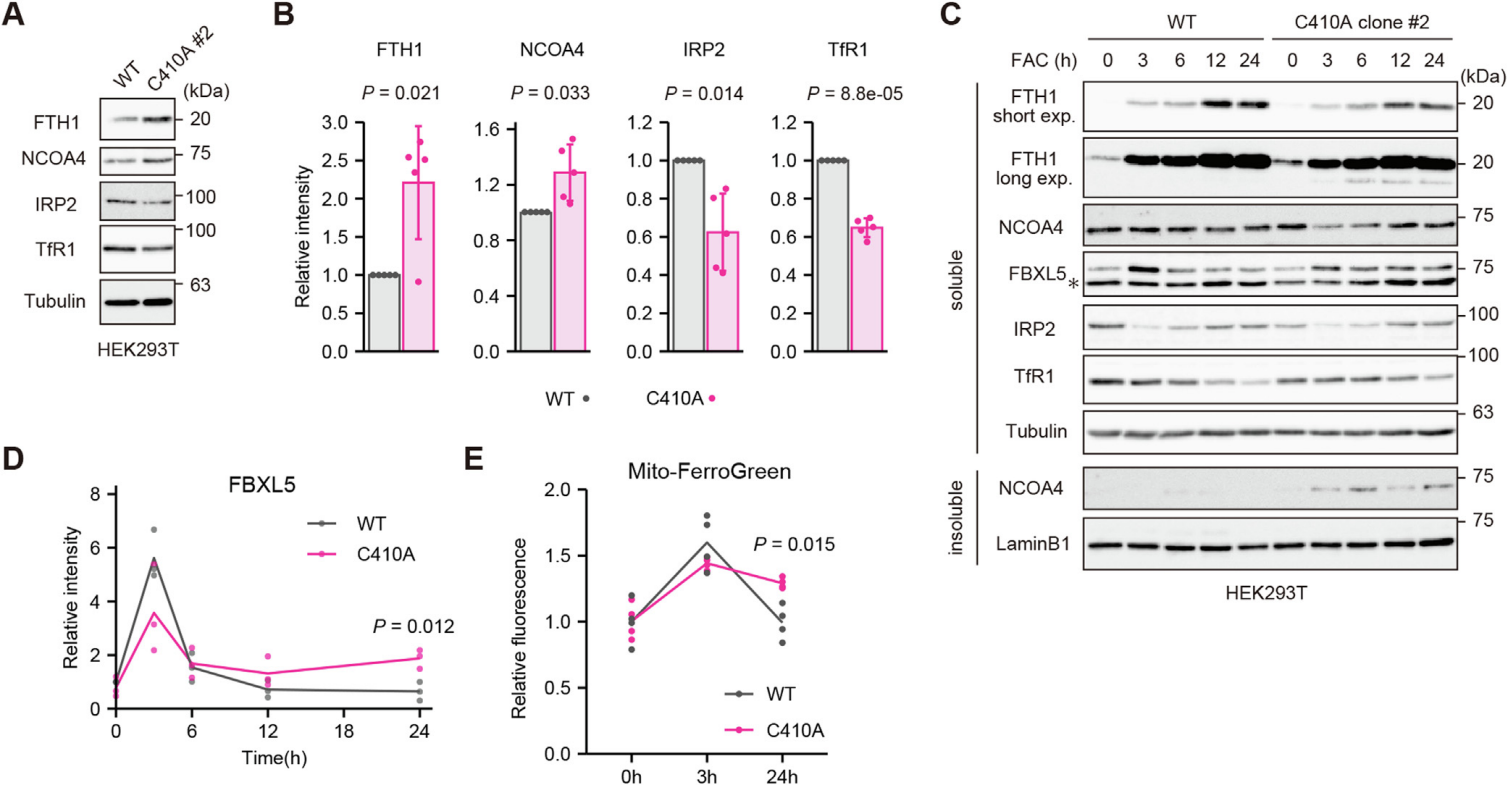
**Noncanonical ferritinophagy alters cellular iron metabolism.***A*, immunoblot analyses of FTH1, NCOA4, IRP2, TfR1, and tubulin in untreated WT HEK293T cells and prime-edited HEK293T cells expressing endogenous NCOA4 C410A (clone #2). Soluble lysates in Triton buffer were subjected to SDS-PAGE. *B*, quantitative analysis of the data shown in (*A*). *p* Values were generated using a one-sample *t* test. *C*, immunoblot analyses of FTH1, NCOA4, FBXL5, IRP2, TfR1, tubulin, and laminB1 in WT and NCOA4 C410A HEK293T cells (clone #2) treated with 25 μg/ml FAC for the indicated times. Soluble and insoluble lysates were subjected to SDS-PAGE and analyzed by immunoblotting with the indicated antibodies. The *asterisk* indicates nonspecific bands. *D*, quantitative analysis of the data for FBXL5 shown in (*C*). Three biological replicates were performed. A Welch’s two-sample *t* test was also performed. *E*, mitochondrial iron levels in WT and NCOA4 C410A HEK293T cells (clone #2) following treatment with 25 μg/ml FAC for 0, 3, or 24 h. The cells were stained with Mito-FerroGreen. *p* Values were generated *via* Welch’s two-sample *t* tests. FAC, ferric ammonium citrate; HEK293T, human embryonic kidney 293T cell line; IRP, iron regulatory protein; TfR1, transferrin receptor 1.

Next, we evaluated the effect of NCOA4-mediated noncanonical ferritinophagy on cellular iron metabolism. The expression level of the iron sensor FBXL5 is increased in iron-replete conditions, and FBXL5 mediates the degradation of IRP2 (25, 26). The amount of IRP2 was higher in WT HEK293T cells than in prime-edited HEK293T cells expressing endogenous NCOA4 C410A (Fig. 4, *A* and *B*), indicating that a defect in noncanonical ferritinophagy in WT cells might induce a response typical of cellular iron deficiency in normal culture medium conditions. By contrast, the observed reduction in the level of TfR1 and increase in the level of ferritin in cells expressing NCOA4 C410A likely counteracted the ferritinophagy-mediated increase in iron delivery by reducing iron uptake and augmenting iron storage (Fig. 4, *A* and *B*). We then added iron to the culture medium of the WT and NCOA4 C410A HEK293T cells and examined time-dependent changes in the expression levels of FBXL5 and iron-related proteins. The expression level of FBXL5 increased rapidly in WT cells and C410A cells at an early time point (3 h) after iron administration (Fig. 4, *C* and *D*). However, the amount of FBXL5 in WT cells was decreased continuously over the observation period (24 h), whereas FBXL5 was maintained at higher levels in C410A cells at later time points. In addition, a faster migrating band representing a partial degradation product of ferritin was observed in C410A cells but not WT cells (Fig. 4*C*). In accordance with the belief that FBXL5 expression reflects the amount of available cellular iron, mitochondrial iron levels were increased in HEK293T C410A cells 24 h after iron administration (Fig. 4*E*). Overall, these results indicate that NCOA4-dependent noncanonical ferritinophagy modulates cellular iron metabolism by providing ferritin-stored iron to cells in steady-state conditions or at late time points after iron administration.

### Oxygen determines the fate of ferritin through biphasic regulation of NCOA4

**Figure 5:**
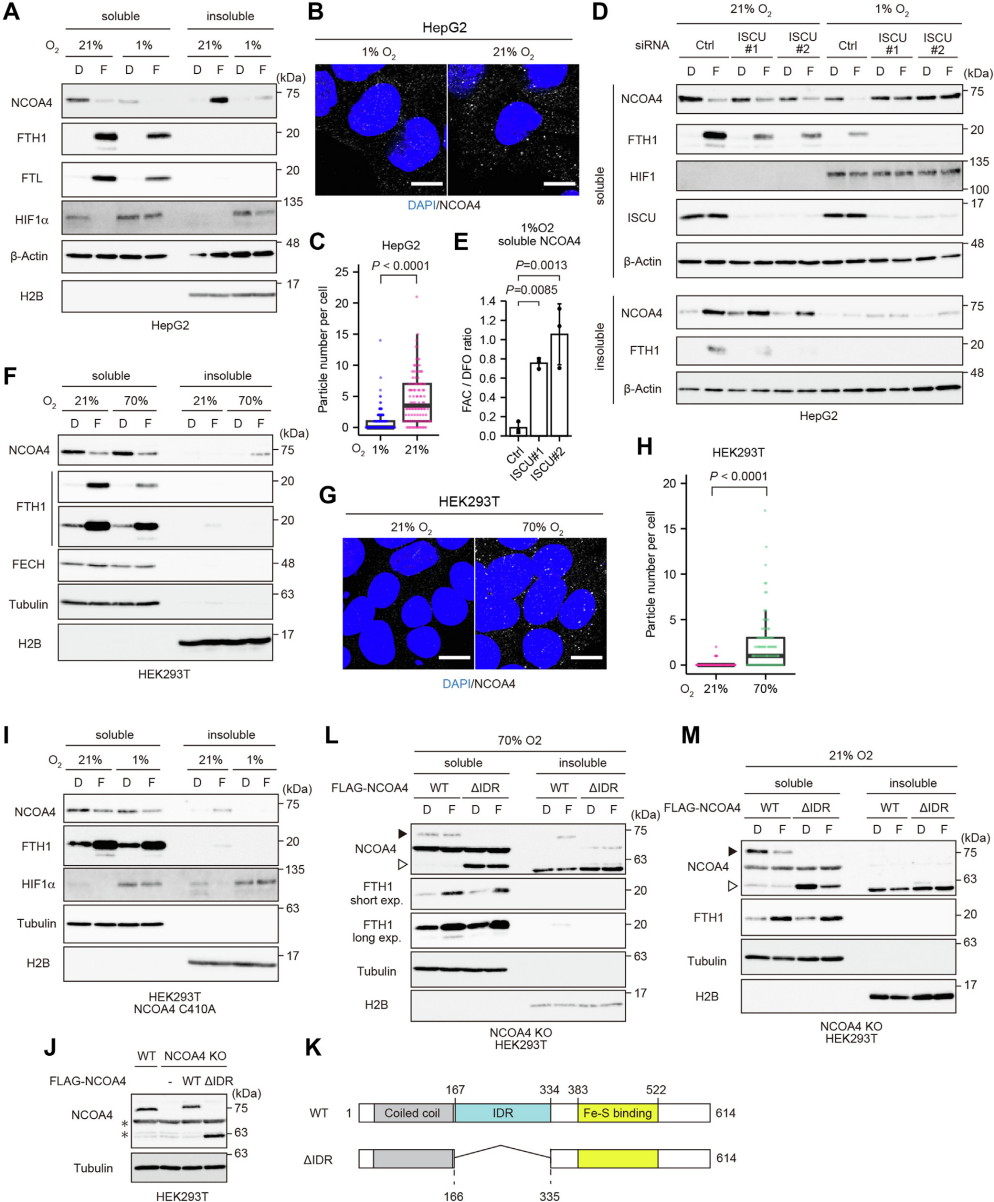
**Oxygen determines the fate of ferritin *via* biphasic regulation of NCOA4.***A*, immunoblot analyses of NCOA4, FTH1, FTL, HIF1α, β-actin, and H2B in HepG2 cells cultured with 20 μM Dfo (D) or 25 μg/ml FAC (F) for 12 h under 21% or 1% O_2_. *B*, fluorescence microscopy images of HepG2 cells cultured with 25 μg/ml FAC for 6 h under 21% or 1% O_2_. The cells were stained with an anti-NCOA4 antibody and DAPI. Representative images are shown. Scale bar represents 10 μm. *C*, quantitative analysis of the particle numbers in the cells described in (*B*). At least 100 cells were quantified from three biological replicates. *D*, immunoblot analyses of NCOA4, FTH1, HIF1, ISCU, and β-actin in HepG2 cells expressing control or ISCU-specific siRNAs. The cells were treated with 20 μM Dfo (D) or 25 μg/ml FAC (F) for 12 h and then harvested. *E*, quantitative analysis of NCOA4 expression in the cells described in (*D*). *p* Values were calculated *via* one-way ANOVA with Dunnett post hoc test. *F*, immunoblot analyses of NCOA4, FTH1, FECH, tubulin, and H2B in HEK293T cells cultured with 20 μM Dfo (D) or 25 μg/ml FAC (F) for 6 h under 21% or 70% O_2_. *G*, fluorescence microscopy images of HEK293T cells cultured with 25 μg/ml FAC for 3 h under 21% or 70% O_2_. The cells were stained with an anti-NCOA4 antibody and DAPI. Representative images are shown. Scale bar represents 10 μm. *H*, quantitative analysis of the particle numbers in the cells described in (*G*). At least 90 cells were quantified from two biological replicates. *I*, immunoblot analyses of NCOA4, FTH1, HIF1α, tubulin, and H2B in NCOA4 C410A HEK293T cells (clone #2) cultured with 20 μM Dfo (D) or 25 μg/ml FAC (F) for 3 h under 21% or 1% O_2_. *J*, immunoblot analyses of NCOA4 and tubulin in WT HEK293T cells, NCOA4 KO HEK293T cells, and NCOA4 KO HEK293T cells stably expressing WT NCOA4 or NCOA4 ΔIDR. The *asterisks* indicate nonspecific bands, part of which overlapped the NCOA4 ΔIDR band. *K*, schematic illustration of the WT NCOA4 and NCOA4 ΔIDR constructs used in (*J*, *L*, and *M*). *L* and *M*, immunoblot analyses of NCOA4, FTH1, tubulin, and H2B in NCOA4 KO HEK293T cells reconstituted with WT NCOA4 or NCOA4 ΔIDR, following culture with 20 μM Dfo (D) or 25 μg/ml FAC (F) for 6 h under 70% O_2_ (*L*) or for 3 h under 21% O_2_ (*M*). The *black arrowhead* indicates WT NCOA4, and the *white arrowhead* indicates NCOA4 ΔIDR, part of which overlapped a nonspecific band. *A*, *D*, *F*, *I*, *L*, and *M*, the cells were fractionated, and the lysates were subjected to SDS-PAGE and analyzed by immunoblotting with the indicated antibodies. *C* and *H*, *p* Values were less than 0.0001 and determined *via* Welch’s two-sample *t* tests. DAPI, 4′,6-diamidino-2-phenylindole; Dfo, deferoxamine; FAC, ferric ammonium citrate; HEK293T, human embryonic kidney 293T cell line; IDR, intrinsically disordered region.

**Supporting Figure S4:**
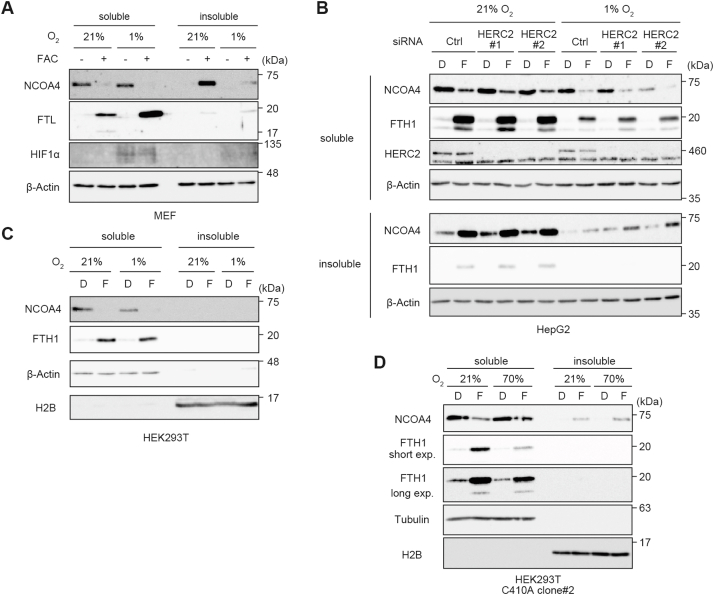
**Oxygen affects the fate of ferritin via regulation of NCOA4.***A*, immunoblot analyses of NCOA4, FTL, HIF1α, and β-actin in MEFs treated with or without 10 μg/ml FAC under 21% or 1% O_2_ for 12 h. *B*, immunoblot analyses of NCOA4, FTH1, HERC2, and β-actin in HepG2 cells expressing a control or HERC2-specific siRNA. The cells were cultured for 2 days and then treated with 20 μM Dfo (D) or 25 μg/ml FAC (F) for 12 h under 21% or 1% O_2_ conditions. *C*, immunoblot analyses of NCOA4, FTH1, β-actin, and H2B in HEK293T cells treated with 20 μM Dfo (D) or 25 μg/ml FAC (F) for 3 h under 21% or 1% O_2_. *D*, immunoblot analyses of NCOA4, FTH1, tubulin, and H2B in NCOA4 C410A HEK293T cells (clone #2) cultured with 20 μM Dfo (D) or 25 μg/ml FAC (F) for 6 h under 21% or 70% O_2_. *A*–*D*, the cells were fractionated, and the lysates were subjected to SDS-PAGE and analyzed by immunoblotting with the indicated antibodies.

Next, we examined whether HERC2-mediated degradation of NCOA4 occurs in cell lines other than HEK293T. In our previous study, we found that NCOA4 preferentially forms insoluble condensates in iron-treated MEFs upon binding to Fe^3+^ (16). Because the cytoplasm has a reducing environment, and oxidation of the ferrous (Fe^2+^) ion to the ferric (Fe^3+^) ion requires oxygen (4, 27), most cytoplasmic iron is thought to be in the reduced bioavailable form (Fe^2+^). In addition, several studies have shown that hypoxia rescues Fe–S availability in cells by protecting Fe–S clusters against molecular oxygen (28, 29). Thus, we hypothesized that oxygen levels might affect the regulation of NCOA4. To investigate this possibility, we cultivated MEFs in hypoxic conditions (1% O_2_) and found that the amount of NCOA4 in the insoluble fraction was lower in iron-replete MEFs at 1% *versus* 21% O_2_ (Fig. S4*A*). As mentioned previously, most NCOA4 was detected in the insoluble fraction of the majority of human-derived cell lines examined here (Fig. 1*A*). Consistent with this finding, immunofluorescence analysis confirmed that NCOA4 formed condensates in iron-treated HepG2 cells (Fig. 5*B*). As expected, NCOA4 was downregulated (Fig. 5*A*), and NCOA4 condensates were not overtly observed (Fig. 5, *B* and *C*) in iron-replete HepG2 cells under hypoxia (1% O_2_). Correspondingly, the faster migrating partial degradation products of ferritin disappeared in hypoxic conditions (Fig. 5*A*).

To examine whether the decrease in NCOA4 expression under hypoxic and iron-replete conditions is mediated *via* recognition of Fe–S cluster–containing NCOA4 by HERC2, we depleted HERC2 expression using specific siRNAs. Under hypoxic conditions, HERC2 depletion substantially increased the amount of insoluble NCOA4 (Fig. S4*B*). Moreover, knockdown of ISCU effectively inhibited NCOA4 degradation under hypoxic and iron-replete conditions, whereas it did not affect iron-dependent NCOA4 condensation under normoxic (21% O_2_) conditions (Fig. 5*D*). These results confirm that Fe–S cluster biogenesis is dispensable for iron-dependent NCOA4 condensation, and that reduced oxygen tension prioritizes Fe–S cluster–dependent NCOA4 degradation in cells. Notably, as observed in HEK293T cells, our findings demonstrate that NCOA4 can be regulated by both Fe–S cluster–mediated degradation and Fe^3+^-mediated condensation in iron-treated HepG2 cells, and the latter was predominant in normoxic conditions.

As described previously, Fe–S cluster–dependent degradation of NCOA4 was predominant in iron-treated HEK293T cells cultured under normoxia (Fig. 2). Since we found that the oxygen tension determined the two fates of NCOA4 in iron-replete HepG2 cells, we next examined the effect of hypoxia on the fate of NCOA4 in HEK293T cells. NCOA4 was degraded in response to iron repletion under both hypoxic and normoxic conditions (Fig. S4*C*). We supposed that the sensitivity to oxygen levels may be altered in HEK293T cells and thus speculated that NCOA4 might form insoluble condensates at high oxygen tension. To test this possibility, we cultivated HEK293T cells under hyperoxic conditions (70% O_2_). A substantial amount of NCOA4 was located in the insoluble fraction of iron-treated HEK293T cells cultured under hyperoxia, and immunofluorescence analysis confirmed that NCOA4 formed condensates in the hyperoxic and iron-replete condition (Fig. 5, *E*–*G*). Moreover, a partial degradation product of ferritin was observed in iron-treated HEK293T cells cultured under hyperoxia (Fig. 5*E*).

**Figure 6:**
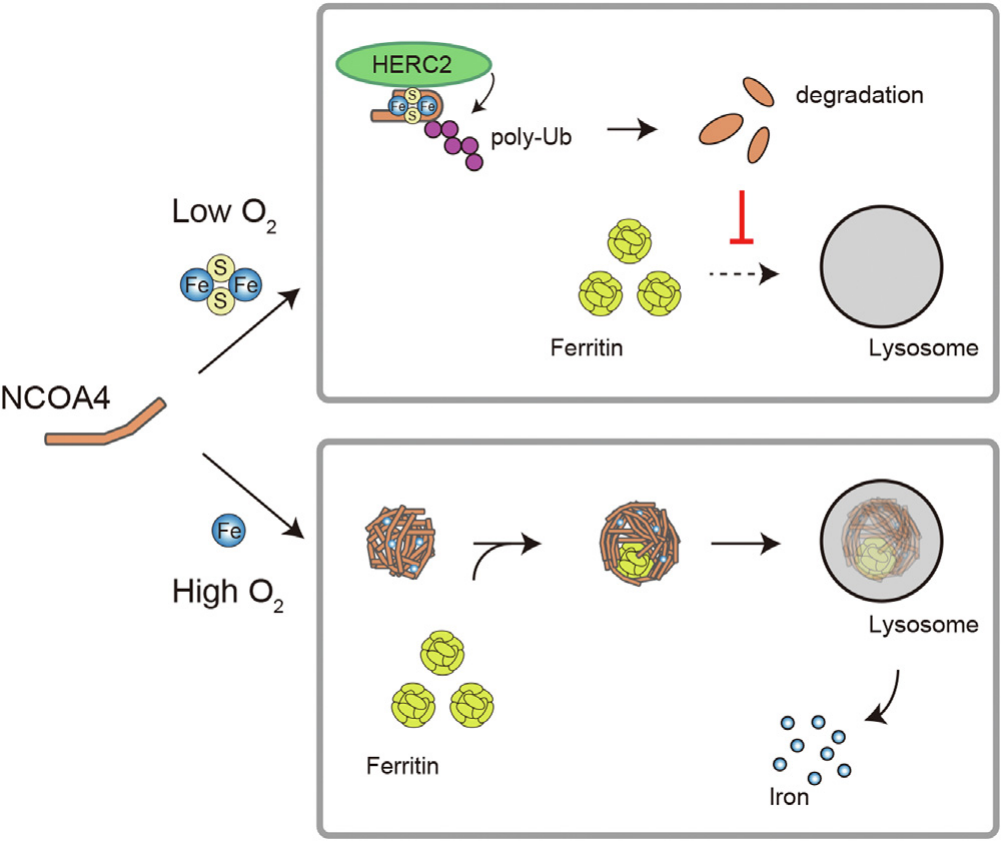
**Schematic summary of the proposed model.** Under low oxygen conditions, an Fe–S cluster is inserted into NCOA4, which is then preferentially recognized by HERC2 and degraded by the proteasome. Degradation of NCOA4 inhibits ferritin autophagy. By contrast, under high oxygen conditions, NCOA4 forms condensates by binding ferric iron, leading to noncanonical autophagy of ferritin and the liberation of iron. Fe–S, iron–sulfur.

To consolidate our model, we examined the effects of the oxygen concentration on the level of the NCOA4 Fe–S binding mutant (C410A) as well as that of the NCOA4 ΔIDR mutant, which cannot bind to ferric iron. NCOA4 C410A was detected in the insoluble fraction of iron-treated HEK293T cells cultured under hyperoxia (70% O_2_) (Fig. S4*D*) but not those cultured under hypoxia (1% O_2_) (Fig. 5*I*). Consistently, a faster migrating FTH1 band disappeared under hypoxia. Cells expressing NCOA4 ΔIDR were generated by reconstituting NCOA4 ΔIDR into NCOA4 KO HEK293T cells (Fig. 5, *J* and *K*). Upon iron administration under hyperoxia, partially degraded ferritin and insoluble NCOA4 were detected in cells expressing WT NCOA4 but not in those expressing NCOA4 ΔIDR (Fig. 5*L*), whereas loss of IDR did not overtly affect NCOA4 degradation under normoxia (Fig. 5*M*). Taken together, these findings demonstrate that while NCOA4 is degraded in an Fe–S cluster–dependent manner in HEK293T cells under low oxygen tension (including normoxia), it forms insoluble condensates, and ferritin is degraded under high oxygen tension. Although the threshold of oxygen tension that determines the fate of NCOA4 differs among cells, and it is unclear why HERC2-mediated degradation operates predominantly in normoxic HEK293T cells (see Discussion section), it appears that oxygen determines the fate of ferritin and iron metabolism in cells *via* differential modulation of NCOA4 (Fig. 6).

## Discussion

The regulation of cellular iron metabolism is essential to avoid iron starvation and toxicity. Cellular iron metabolism is thought to be regulated chiefly by the FBXL5–IRP system. IRPs control the production of ferritin and TfR1 by binding to IREs in their transcripts under iron-deficient conditions, and FBXL5 degrades IRP2 under iron-replete conditions (11, 12). To achieve tight regulation of iron availability, the amount of ferritin is also regulated by NCOA4-mediated autophagy. In response to iron depletion, ferritin is delivered to lysosomes *via* NCOA4-mediated macroautophagy, promoting the release of stored iron (14, 15). In addition, to prevent iron deficiency caused by excessive iron storage in prolonged iron-replete conditions, ferritin is delivered to lysosomes *via* a noncanonical autophagy pathway mediated by iron (Fe^3+^)-induced NCOA4 condensation (16). Although condensed NCOA4 is sequestered away from ferritin to ensure storage of excess iron in the early phase of iron repletion, we found here that NCOA4 containing an Fe–S cluster can also be sequestered from ferritin *via* ubiquitin-mediated degradation in some settings. In addition, we found that the oxygen level determines the fate of ferritin by switching the mode of NCOA4 regulation; while high oxygen levels provoke ferritinophagy by inducing binding of Fe^3+^ to NCOA4, low oxygen levels facilitate the insertion of an Fe–S cluster into NCOA4, rendering it susceptible to HERC2-mediated degradation and inhibiting ferritin autophagy (Fig. 6). Although it is technically challenging, clarifying the metalation status of NCOA4 in both the soluble and insoluble fractions of lysates would deepen our understanding of the complex iron-mediated regulation of NCOA4.

Oxygen is also involved in FBXL5-mediated degradation of IRP2 (25, 26). The amount of IRP2 is increased under low oxygen levels because of the instability of FBXL5, leading to reduced ferritin synthesis (25, 26, 30). This finding indicates that hypoxia resets the threshold of the FBXL5–IRP system to allow cells to accumulate larger amounts of available iron than they do under normoxia. However, in the case of NCOA4-mediated ferritin turnover, hypoxia inhibits ferritin turnover, which leads to a reduction in the level of available iron by increasing iron sequestration. Although the FBXL5–IRP system and NCOA4–ferritin axis appear to have opposing functions under various oxygen conditions, coregulation of the synthesis and degradation of ferritin by the two systems would enable tight regulation of cellular iron homeostasis. Therefore, the NCOA4–ferritin axis is another mechanism for controlling cellular iron homeostasis that acts co-operatively with the FBXL5–IRP system in response to oxygen levels.

The Fe–S cluster participates in several essential biological processes, such as electron transport in mitochondrial oxidative phosphorylation, and DNA synthesis and repair in the nucleus (19, 20, 21). In addition, some iron-regulated proteins sense the cellular iron status *via* the Fe–S cluster. For example, the activities of the transcriptional activators Aft1 and Aft2 (Aft1/2), which are the central regulators of iron metabolism in *Saccharomyces cerevisiae* (31, 32, 33, 34), are regulated by the Fe–S cluster. Aft1/2 activate the transcription of genes that comprise the iron regulon only under iron-limiting conditions, and binding of the Fe–S cluster to Aft1/2 induces their dissociation from target promoters under iron-replete conditions (35, 36). The Fe–S cluster is also involved in regulation of the FBXL5–IRP system. Ligation of the 2Fe–2S cluster to FBXL5 is a prerequisite for its ability to recognize and degrade IRPs in response to iron repletion (22). However, IRP1 is also regulated by the Fe–S cluster in another way. IRP1 binds to its target proteins containing IREs in iron-depleted condition, whereas it loses its IRE-binding activity and functions as a cytosolic aconitase by harboring a 4Fe–4S cluster in iron-replete conditions (37). Here, we found that insertion of an Fe–S cluster into NCOA4 leads to ubiquitin-mediated degradation of the protein (Fig. 2). Because the Cys residues in NCOA4 gripping the Fe–S cluster are evolutionally conserved from fish to human (Fig. 2*A*), the sensing mechanism may play an important role in maintaining iron homeostasis in various organisms. A recent study reported that NCOA4-dependent ferritinophagy prioritizes the supply of iron to Fe–S cluster synthesis to maintain pancreatic cancer proliferation (38). Thus, the Fe–S cluster–mediated iron-sensing ability of NCOA4 may have important roles in some pathological situations.

We demonstrated previously that NCOA4 forms insoluble condensates by binding to Fe^3+^, and the condensates direct ferritin to lysosomes *via* noncanonical autophagy in response to prolonged iron repletion (16). Hemosiderin is a pathological marker of iron overload found in several organs, including the liver. Since hemosiderin is formed by the aggregation of denatured ferritin in lysosomes (39), the delivery of ferritin to lysosomes under iron-replete conditions should increase hemosiderin production. Here, we found that ferritin degradation occurs preferentially under high oxygen conditions (Fig. 5). The oxygen tension is not uniform in the liver. Although the liver consists of hexagonal lobules, it is divided into three distinct functional zones, in which the oxygen concentration is altered gradually depending on the distance from the hepatic artery (40). Based on our observations, it could be hypothesized that hemosiderin is localized mainly in the zone with high oxygen tension. Indeed, previous studies have shown that hemosiderin is located predominantly in hepatocytes in the peripheral region of the lobules, which is the area closest to the hepatic arteries, and the amount of hemosiderin decreases toward the center of the lobules (41, 42). Considering these findings, it is possible that iron overload promotes NCOA4-mediated ferritin delivery to lysosomes in an oxygen tension–dependent manner.

Iron is often utilized as an electron carrier, especially in enzymatic reactions catalyzing oxidation or reduction in organisms (43). As mentioned previously, Fe–S clusters are vulnerable to molecular oxygen (44). In addition, iron functions as the catalytic center of enzymes that utilize oxygen as substrates, such as dioxygenases (43). These enzymes can be damaged by electron transfer during enzymatic reactions. Therefore, to replace the destroyed Fe–S cluster proteins and damaged enzymes, iron demand is likely to be higher in cells with high oxygen tension. Indeed, our findings demonstrate that high oxygen concentration induces ferritinophagy (Fig. 5), and enhancement of ferritinophagy paradoxically increases ferritin expression *via* the compensatory actions of the FBXL5–IRP system (Fig. 4, *A* and *B*). Thus, in cells under high oxygen tension, it seems necessary to deliver available iron *via* ferritin autophagy, and to simultaneously protect cells against iron toxicity and the associated oxidative stress by increasing the amount of ferritin, a process that is achieved by coordinated regulation of the FBXL5–IRP system and the NCOA4–ferritin axis. In our experiments, the threshold of oxygen tension varied between cell lines, which might be attributable to differences in the intracellular reductive milieu, destruction of Fe–S clusters, and/or the generation of ferric iron.

The amount of available iron is a critical component determining the sensitivity of ferroptosis (6, 7), and loss of NCOA4 suppresses ferroptosis in some settings (45, 46). Previous studies demonstrated that inhibition of Fe–S cluster synthesis by NFS1 depletion predisposes cancer cells to ferroptosis (28, 47). In addition, inhibition of Fe–S cluster biosynthesis increases the iron demand (48), which may enhance NCOA4-mediated ferritinophagy in iron-rich conditions and result in augmentation of ferroptosis. Noncanonical ferritinophagy functions to overcome relative iron starvation in iron-replete conditions, which may be caused by physiological and pathological settings such as high oxygen tension. Therefore, NCOA4 plays a role in ferroptosis by releasing excess iron from ferritin *via* ferritinophagy in some pathological settings. Further studies are required to understand the relationships between ferroptosis and NCOA4-mediated ferritinophagy in various oxygen level conditions *in vivo*.

## Experimental procedures

### Plasmids

The open reading frame of human *NCOA4* was cloned by RT–PCR using mRNA isolated from HEK293 cells. The following variants were generated: C404S, C410S, C416S, C418S, C422S, and 4CS (C404S, C410S, C416S, and C422S). The human *HERC2* complementary DNA was a gift from Dr Tomohiko Ohta (St Marianna University School of Medicine). The complementary DNAs were ligated to the appropriate epitope-tag sequences and then cloned into the pcDNA3.2, pMX IRES puro FLAG, and pGEX6p-1 vectors. An sgRNA targeting *NCOA4* (5′-AAAAGGGTCATTACCTCTCC-3′) was cloned into the PX458 plasmid (Addgene) to produce NCOA4 KO cells.

### Antibodies and reagents

The following antibodies were used in this study: anti-ferritin (Sigma; catalog no.: F6136; Western blotting [WB], 1:2000 dilution), anti-FTH1 (Santa Cruz; catalog no.: sc-376594; WB, 1:2000 dilution), anti-NCOA4 (Santa Cruz; catalog no.: sc-373739; WB, 1:300 dilution), anti-NCOA4 (Invitrogen; catalog no.: PA5-96398; immunofluorescence, 1:500 dilution), anti-HERC2 (BD Biosciences; catalog no.: 612366; WB, 1:2000 dilution), anti-TAX1BP1 (Abcam; catalog no.: ab176572; WB, 1:5000 dilution), anti-IRP2 (our laboratory; WB, 1:1000 dilution), anti-IRP2 (Cell Signaling Technology; catalog no.: 37135; WB, 1:1000 dilution), anti-FBXL5 (Santa Cruz; catalog no.: sc-390102; WB, 1:1000 dilution), anti-TfR1 (Invitrogen; catalog no.: 13-6890; WB, 1:3000 dilution), anti-ISCU (our laboratory; WB, 1:2000 dilution), anti-HIF1 (our laboratory; WB, 1:2000 dilution), anti-FECH (Santa Cruz; catalog no.: sc-377377; WB, 1:1000 dilution), anti-β-actin (Sigma; catalog no.: A5316; WB, 1:15,000 dilution), anti-tubulin (our laboratory, WB, 1:5000 dilution), anti-FLAG (Sigma–Aldrich; catalog no.: F7425; immunoprecipitation), anti-DDDDK (MBL; catalog no.: PM020; WB, 1:3000 dilution), anti-myc (Millipore; catalog no.: 05-724; WB, 1:2000 dilution), anti-H2B (Millipore; catalog no.: 07-371; WB, 1:3000 dilution), anti-laminB1 (Abcam; catalog no.: 16048; WB, 1:3000 dilution), horseradish peroxidase–linked anti-mouse immunoglobulin G (Cell Signaling Technology; catalog no.: 7076; WB, 1:10,000 dilution), and horseradish peroxidase–linked anti-rabbit immunoglobulin G (GE Healthcare; catalog no.: NA934; WB, 1:10,000 dilution).

The following reagents were used in this study: FAC (Sigma–Aldrich; catalog no.: F5879), deferoxamine mesilate (Desferal; Novartis; catalog no.: V03AC01), E64d (Peptide Institute; catalog no.: 4321-v), and pepstatin A (Peptide Institute; catalog no.: 4397-v).

### Cell lines and cell culture

MEFs and the HepG2, U2OS, HeLa, HEK293T, RD4, UOK111, and MCF7 cell lines were cultured in Dulbecco's modified Eagle's medium (Sigma) supplemented with 10% fetal bovine serum (Sigma), 100 IU/ml penicillin, and 100 μg/ml streptomycin and were maintained at 37 °C under 7.5% CO_2_ in humidified air. A multigas incubator (APM-30DR; Astec) was used to culture cells under 1% O_2_ conditions. For culture under hyperoxia, cell culture plates or dishes were placed into a plastic bag (Tedlar bag; Ohmi Odo-Air Service), and the bag was sealed completely with a clip. The air was then flushed out, and the bag was refilled with a high oxygen gas mixture (70% O_2_, 25% N_2_, and 5% CO_2_). Subsequently, the gas mixture was flushed out, and the step was repeated twice. Finally, the bag was filled with the high oxygen gas mixture and placed in a 37 °C incubator.

### Plasmid DNA and siRNA transfection

Appropriate pcDNA3.2 plasmids were transfected into HEK293T cells using PEI MAX (Polysciences). The siRNAs were transfected into cells using Lipofectamine RNAiMAX. Plasmid DNA and siRNAs were cotransfected into cells using Lipofectamine 2000.

### Generation of the NCOA4 C410A HEK293T cell line

HEK293T cells were transfected with the PE2 plasmid, epegRNA plasmid, sgRNA plasmid (for PE3), and pcDNA3.2 GFP plasmid (for sorting) using Lipofectamine 2000. After 24 h, GFP-positive cells were selected using a FACS Aria III cell sorter. Fifty cells were seeded into a 10 cm dish, and single clones were obtained using cloning rings. The C410A mutation was confirmed by sequencing of PCR products generated from genomic DNA.

### Generation of CRISPR/Cas9-mediated KO cells

To generate NCOA4 KO cells, a PX458 plasmid encoding a sgRNA targeting *NCOA4* was transfected into HEK293T cells using PEI MAX. After 24 h, GFP-expressing cells were isolated using an FACS Aria III sorter (BD Biosciences), and single clones were obtained using cloning rings.

### Retroviral infections and generation of stable cell lines

Appropriate pMXs plasmids and the pVSV-G plasmid were transfected into GP2-293 packaging cells (Takara Bio) using PEI MAX. After 48 h, retrovirus in the culture medium was collected and passed through a 0.45 μm filter. NCOA4 KO cells were infected with the retrovirus in the presence of polybrene (10 μg/ml) for 16 h. The infected cells were selected using puromycin.

### Cell lysis and fractionation

For preparation of total cell lysates, cells were lysed in 1× sample buffer (50 mM Tris–HCl, pH 6.8, 2% SDS, 10% glycerol, 0.1% bromophenol blue, and 100 mM DTT). The cell lysate was sonicated to shear DNA and then boiled at 95 °C for 10 min.

To prepare soluble and insoluble samples, cells were lysed in Triton buffer (1% Triton X-100, 50 mM Tris–HCl, pH 8.0, and 150 mM NaCl) supplemented with 2 mM PMSF and protease inhibitor cocktail (Roche). After incubation on ice for 20 min, the soluble extract was collected after centrifugation at 20,000*g* for 20 min at 4 °C. After the addition of 4× sample buffer, the sample was boiled at 95 °C for 5 min. The insoluble pellet was washed once with Triton buffer and resuspended in 1× sample buffer. The insoluble sample was then sonicated and boiled at 95 °C for 10 min.

### Immunoblotting

Samples were resolved by SDS-PAGE and transferred to a polyvinylidene fluoride membrane. After blocking in Tris-buffered saline containing 0.1% Tween-20 and 5% (w/v) nonfat dry milk, membranes were incubated with the appropriate primary antibodies, followed by the corresponding secondary antibodies. Bands were visualized by enhanced chemiluminescence, and the signals were detected on a LAS4000 mini-instrument (GE Healthcare).

### Immunoprecipitation

To detect the interaction between NCOA4 and HERC2, an anti-FLAG antibody was incubated with the Triton X-100 soluble cell lysate for 90 min at 4 °C. After the addition of Protein A beads, the sample was incubated with rotation for 60 min at 4 °C, followed by four washes with Triton buffer and two washes with 20 mM Tris–HCl (pH 8.0). Immunoprecipitated proteins were denatured by the addition of 2× sample buffer and boiling at 95 °C for 5 min.

An anti-ferritin antibody was used to isolate endogenous ferritin from Triton X-100 soluble cell lysates. An anti-FLAG antibody was used as a control. Dynabeads Protein A (Invitrogen) conjugated with the antibodies were added to the soluble lysates, and the samples were incubated with rotation for 60 min at 4 °C, followed by four washes with Triton buffer. Immunoprecipitated proteins were denatured by the addition of 2× sample buffer and boiling at 95 °C for 5 min.

### Protein expression and purification

Proteins were expressed in and purified from *Escherichia coli* strain BL21-CodonPlus (DE3)-RIPL. When WT GST-NCOA4 (amino acids 383–522), and its mutants were purified, bacteria were grown in LB medium supplemented with 121 μg/ml Cys, 25 μg/ml FAC, and 50 μg/ml ampicillin. The bacteria were grown to an absorbance o 0.6 at 600 nm for 30 °C before induction with 100 μM IPTG at 16 °C for 18 h. Next, the bacteria were collected by centrifugation and lysed by sonication in GST lysis buffer (20 mM Tris–HCl, pH 7.5, 150 mM NaCl, and 1 mM DTT) supplemented with 2 mM PMSF and protease inhibitor cocktail. The sample was centrifuged at 23,000*g* for 30 min at 4 °C, and the supernatant was mixed with Glutathione Sepharose 4 Fast Flow (GE Healthcare) for 1 h at 4 °C. The beads were washed with GST lysis buffer, and GST protein was eluted with glutathione buffer (20 mM Tris–HCl, pH 8.0, 200 mM NaCl, and 20 mM glutathione). The eluted sample was desalted using a PD-10 column and GST lysis buffer.

When His-HERC2 (amino acids 2540–2700) was purified, bacteria were grown in LB medium supplemented with 50 μg/ml ampicillin. The bacteria were grown to an absorbance of 0.6 at 600 nm at 30 °C before induction with 100 μM IPTG at 16 °C for 18 h. Next, the bacteria were collected by centrifugation and lysed by sonication in His lysis buffer (20 mM Tris–HCl, pH 8.0, 300 mM NaCl, and 10 mM imidazole) supplemented with 2 mM PMSF, 2 mM 2-ME, and protease inhibitor cocktail. The sample was centrifuged at 23,000*g* for 30 min at 4 °C, and the supernatant was mixed with nickel–nitrilotriacetic acid beads (QIAGEN) for 1 h at 4 °C. The beads were then washed three times with wash buffer 1 (20 mM Tris–HCl, pH 8.0, 300 mM NaCl, and 20 mM imidazole) and twice with wash buffer 2 (20 mM Tris–HCl, pH 8.0, 300 mM NaCl, and 30 mM imidazole). The His-tagged protein was eluted with His elution buffer (20 mM Tris–HCl, pH 8.0, 300 mM NaCl, and 300 mM imidazole), and the eluted sample was desalted using a PD-10 column and a buffer comprising 20 mM Tris–HCl, pH 7.5, 150 mM NaCl, and 1 mM DTT.

### UV–visible absorption spectrometry

UV–visible absorption spectra were measured at a range of 250 to 800 nm using a V-750 UV–Visible Spectrophotometer (JASCO) at room temperature. The protein samples (50 μl of 0.8–2.0 mg/ml) were in buffer containing 20 mM Tris–HCl, pH 7.5, 150 mM NaCl, and 1 mM DTT.

### Pulldown assay

GST or GST-tagged proteins (2 μg) and His-HERC2 (amino acids 2540–2700) (2.8 μg) were mixed in 800 μl of binding buffer (20 mM Tris–HCl, pH 7.5, 150 mM NaCl, 0.1% Triton X-100, and 1 mM DTT). After the addition of 10 μl of Glutathione Sepharose 4 Fast Flow beads, the samples were incubated for 1 h at 4 °C with rotation. The beads were then washed four times with binding buffer and twice with PBS containing 1 mM DTT. Proteins were eluted with 2× sample buffer and boiled at 95 °C for 5 min.

### Immunocytochemistry

Cells were seeded onto collagen-coated cover glasses at least 24 h before use. The cells were then fixed with 4% formaldehyde in PBS for 20 min at room temperature. The fixed cells were washed three times with PBS and then permeabilized for 3 min with 0.1% Triton X-100 in PBS. After washing three times with PBS, the cells were incubated with blocking buffer (2% bovine serum albumin in PBS) for 1 h at room temperature. The samples were then incubated with the primary antibodies in blocking buffer for 1 h at room temperature or overnight at 4 °C, washed three times in PBS, and incubated with the secondary antibodies in blocking buffer for 1 h at room temperature. Subsequently, the samples were washed again with PBS and then stained with 0.1 μg/ml 4′,6-diamidino-2-phenylindole in PBS for 5 min at room temperature. Finally, the samples were washed three times with PBS and mounted with Fluoro-KEEPER Antifade Reagent (Nacalai Tesque). Confocal fluorescence images were acquired with an IX81 inverted microscope (Olympus) equipped with an FV1000 confocal imaging system (Olympus) and a 60×/1.42 numerical aperture oil objective lens (PlanApo N 60X; Olympus). Images were analyzed and quantified using the ImageJ (NIH) and R software packages (R Core Team).

### Measurement of mitochondrial iron

Cells were seeded into collagen-coated glass-bottomed dishes and treated with FAC as indicated. After washing with PBS, the cells were incubated with 5 μM Mito-FerroGreen (Dojindo) in Hanks’ balanced salt solution at 37 °C for 30 min and then washed twice with Hanks’ balanced salt solution. Confocal fluorescence images were acquired as mentioned previously. Images were analyzed and quantified using the ImageJ and R software packages.

### Statistical analysis

All bar graphs show the mean ± SD. In boxplots, bold lines indicate the median, boxes indicate the interquartile range (25–75th percentile), and whiskers indicate 1.5 times the interquartile range. Statistical significance was analyzed by one-sample *t* test, Welch’s two-sample *t* test, one-way ANOVA with Dunnett post hoc test, or Kruskal–Wallis ANOVA with Dunn’s multiple comparison test, as indicated in each figure legend. Data visualization and statistical analyses were performed using R software packages. All experiments were performed at least twice, and consistent results were obtained.

## Data availability

This study includes no data deposited in external repositories.

## Supporting information

This article contains supporting information.

## Conflict of interest

The authors declare that they have no conflicts of interest with the contents of this article.
